# Characterization of cornea donors at a tissue center in the city of Medellin, Colombia

**DOI:** 10.1007/s10561-023-10124-x

**Published:** 2023-12-23

**Authors:** Juan Camilo Cadavid Usuga, María Isabel Maya Naranjo, Sara Mesa Mesa, Ana Isabel Rivera, María Paula Hincapié, María Fernanda Granados, Catalina Betancur, Jorge Hernando Donado Gomez

**Affiliations:** 1 Department of Ophthalmology, Pontifical Bolivarian University, Medellín, Colombia; 2 Ophthalmology Research Group, Pontifical Bolivarian University, Medellín, Colombia; 3 Eye Bank Department, Colombian Red Cross, Antioquia Section, Medellín, Colombia; 4 Research Department, Pablo Tobón Uribe Hospital, Medellín, Colombia; 5 Pontifical Bolivarian University, Medellín, Colombia; 6 Girardota, Colombia 051030

**Keywords:** Corneal transplantation, Eye banks, Tissue donors, Cornea

## Abstract

The cornea transplant is considered the most frequently performed type of transplant in the world, with a demand that has been increasing in recent years. An observational descriptive study was conducted, focusing on the ocular tissue extracted from cadaveric donors from January 2019 to December 2021 at the Red Cross Eye Bank in Medellin, Colombia. This is the first epidemiological characterization of corneal donor tissues within the eye banks of our city, where high rates of violence-related deaths explain that tissue donors are mostly young individuals. This, in turn, results in excellent counts of endothelial cells and tissue viability in their microscopic studies. Additionally, there are lower rates of discarded tissues compared to similar studies.

## Introduction

Corneal blindness is a significant cause of avoidable blindness and ranks as the third leading cause of blindness worldwide, following cataracts and glaucoma (Gain et al. 2016). The most common causes of unilateral and bilateral corneal blindness include childhood keratitis (36.7%), trauma (28.6%), and adult-onset keratitis (17.7%) (Gupta et al. 2018; Singh et al. 2019).

Corneal transplantation relies on the availability of donor tissues, a factor that has significantly impacted the transplantation rate worldwide. Based on the current availability of donor tissues and demand rates, it is estimated that 270,000 donor tissues are needed annually to perform 100,000 corneal transplants in India alone, posing a challenge for public health specialists and the healthcare system at large (Gupta et al. 2018). The aim of this study is to describe the demographic and clinical characteristics of corneal donors at a high-volume tissue center in the city of Medellin from 2019 to 2021.

## Methodology

We conducted a descriptive observational study focusing on ocular tissue extracted from cadaveric donors at the Red Cross Eye Bank in Medellin, Colombia, from January 2019 to December 2021. Data collection was performed by the study investigators using a database designed by the research group, which included measurements of sociodemographic variables, macroscopic characteristics, and microscopic examination of donor tissues, following a pilot test. Absolute and relative frequencies were used for qualitative variables, while measures of central tendency and dispersion were employed for quantitative variables. Data analysis was conducted using the statistical software Epidat version 4.2, a program for epidemiological data analysis (Consellería de Sanidade and de Galicia 2016). The researchers adhered to the Declaration of Helsinki version 2013 for research involving human subjects and obtained approval from the Ethics Committee for Research at Pontifical Bolivarian University.

## Results

Between January 2019 and December 2021, at the Red Cross Eye Bank in Medellin, Colombia, a total of 1050 corneas were collected from 528 patients. Of these, 65 (6%) were discarded. The tissues were obtained from patients who had passed away in various locations, with 57.4% from public places, 25% from healthcare facilities, 16.9% from their place of residence, 0.2% from their workplace, and 0.6% from unspecified locations. Among the donors, 80% were male, with an average age of 36.95 ± 14 years.

The causes of death among donors were as follows: firearm injuries (27.6%), traffic accidents (19.7%), natural causes (19.7%), suicide (13.3%), trauma (8.9%), stab wounds (7.8%), intoxication (2.8%), and airway obstruction (0.2%).

Out of all the tissues included in the study, only 65 corneas (6%) were discarded. The primary reason for discarding was infectious reactivity to Syphilis (47.69%), HIV (24.61%), Hepatitis C (23.30%), Hepatitis B (9.23%) followed by reactivity to COVID-19 (3.07%) and the macroscopic appearance of the corneal tissue outside the parameters established by the Eye Bank at the time of recovery (3.07%).

Among the corneas suitable for donation, 99.8% were classified as excellent quality by the receiving surgeons. Microscopic characteristics of the tissues are described in Table 1.

**Table 1:** Microscopic characteristics of tissues in 1050 corneas

	Frequency N (%)
*Epithelial defect*	
Without defect	754 (71.8)
< 25%	188 (17.9)
25–50%	42 (4)
> 50%	3 (0.2)
No information	1 (0.1)
Discarded	65 (6)
*Epithelial edema*	
Without edema	416 (39.6)
< 25%	563 (53.6)
25–50%	7 (0.6)
> 50%	1 (0.1)
No information	1 (0.1)
Discarded	65 (6)
*Opacity*	
No opacities	976 (92.9)
Peripheral opacities	3 (0.3)
Central opacities	4 (0.4)
No information	4 (0.4)
Discarded	65 (6)
*Foreign bodies*	
No foreign bodies	982 (93.5)
Present	1 (0.1)
No information	4 (0.4)
Discarded	65 (6)
*Descemet's folds*	
Absent	6 (0.57)
Sparse	978 (93.1)
Plentiful	2 (0.2)
No information	1 (0.1)
Discarded	65 (6)
*Guttas*	
Absent	985 (93.8)
Central	1 (0.1)
Peripheral	0 (0)
No information	1 (0.1)
Discarded	65 (6)
*Arcus senilis*	
Absent	860 (81.9)
Present	126 (12)
No information	1 (0.1)
Discarded	65 (6)
*Others*	
Pterygium	16 (1.5)
Peripheral lacerations	2 (0.2)
*Coefficient of variation (polimegatism)*	
< 25%	6 (0.57)
26–50%	971 (92.4)
> 50%	8 (0.8)
No information	2 (0.2)
Discarded	65 (6)
*Hexagonality (polymorphism)*	
< 25%	1 (0.1)
26–50%	329 (31.3)
> 50%	655 (62.3)
No information	4 (0.2)
Discarded	65 (6)

## Discussion

Corneal transplantation is considered the most frequently performed type of transplant worldwide, with a growing demand in recent years. This procedure serves as an effective therapeutic option for severe corneal diseases, significantly impacting the visual prognosis of recipient patients (Gain et al. 2016). The success rate of corneal transplantation depends on various variables, including tissue transportation, processing, and storage protocols, thorough evaluation of the histological and anatomical characteristics of donor corneal tissue, and proper collection of medical histories from the cadaveric donor population, among others (Gain et al. 2016; Gupta et al. 2018; Singh et al. 2019). This justifies the importance of conducting a demographic characterization of our population based on cadaveric donors to directly influence the development of local and national strategies aimed at optimizing donor protocols and reducing the incidence of corneal blindness.

According to epidemiological data presented by the Pan American Association of Eye Banks (APABO) and records from the Pan American Health Organization (PAHO) consensus, a significant deficit in the number of corneal donors exists in Latin America and the Caribbean following the declaration of the COVID-19 pandemic (Association and of Eye Banks - APABO 2022; PAHO.org 2022). This is due to the additional strict criteria established by the international community of Eye Banks for donor selection. This situation explains the drastic reduction in the number of corneal transplants in different populations in 2020. For example, Brazil went from a total of 3412 transplants in the first quarter of 2020 to 286 transplants in the following 2 months (Association and of Eye Banks - APABO 2022).

In Colombia, according to the latest report from the National Institute of Health (INS) in February 2022, there were 477 potential donors under the brain death protocol, resulting in 47 actual donors, and 389 alerts under the cardiac arrest protocol, leading to 239 tissue donors. As of February, there were 6 corneal emergency cases (National Institute of Health 2022). The transplant rate in Bogotá decreased by 44% in 2020 compared to the previous year. However, there was a recovery in the rate, reaching 44.8 transplants per million inhabitants in 2021. Regarding corneal tissue, as of March 31, 2022, a total of 303 successful transplants were performed, with a waiting list of 209 patients over the past year (Foxley 2009).

The role of eye banks is crucial in ensuring the proper collection, transportation, processing, and storage of tissues. An ideal collection time of less than 12 h has been proposed to reduce the infection rate related to grafts and ensure better graft quality. Eye banks must ensure the quality of corneal tissue by maintaining proper asepsis during tissue extraction, considering that any source of infection can lead to complications with a poor visual prognosis, such as graft infection and endophthalmitis (Singh et al. 2019). Graft failure has been reported in up to 18.4%, with corneal infection being the most common cause (50%) (Sousa and Sousa 2018).

As described in other studies, the age range of corneal tissue donors in a cohort in Brazil from 2014 to 2017 was 40 ± 15.9 years, in contrast to our study, where the average age was 36.95 ± 14 years. This lower age in our study may be due to violent deaths, with the primary cause of death in our region being firearm injuries (27.5%), followed by traffic accidents (19.7%) and natural causes (19.7%). In contrast, the mentioned study population had cardiovascular disease as the leading cause of death in 25% of cases, followed by stab wounds in 20% and polytrauma in 14% (Victer et al. 2019).

Various clinical conditions make a corneal donor unfavorable due to the risks associated with the tissue. Consequently, donor selection protocols have been developed in eye banks worldwide. Thus, conditions such as transmissible infectious diseases like hepatitis B, C, and HIV, disseminated bacterial and fungal infections, ocular abnormalities like keratoconus, glaucoma, anterior uveitis, and refractive surgery, neoplasms like retinoblastoma, and other conditions such as hepatitis, jaundice, and prion diseases have been included as contraindications for donors (Castillo et al. 2020). In our cohort, only 65 corneas were discarded (6% of them), with 93% discarded due to infectious reactivity (HIV, syphilis, and hepatitis B), 3.17% each due to reactivity to COVID-19, and macroscopic appearance. In our protocol, all corneas with central opacities, foreign bodies, arcus senilis > 3 mm, polymegathism > 76%, and hexagonality < 50% were discarded. In contrast, other studies discarded 23.9% of tissues, with the main reasons being serological reactivity or indetermination in 68.3%, stromal infiltrate in 37.2%, undetermined cause of death in 13.8%, and sepsis in 9.3%.

Similarly, various sociodemographic characteristics can make one tissue more optimal than another. Different research studies have demonstrated that donor age is not the most important factor in determining tissue quality. Theoretically, younger donors should have higher endothelial cell densities; however, this depends on the patient's comorbidities and personal history. Numerous corneas from donors over the age of 60, following endothelial status evaluation, have been deemed suitable for the procedure (Castillo et al. 2020) and (Tanyildiz et al. 2021). In our cohort, the average age was found to be 36.95 ± 14 years, which is below the average reported in the literature, due to violent deaths in our region.

Our study stands out for its significant sample size and a 3-year follow-up as a strength. Additionally, it represents the first epidemiological characterization of corneal donor tissues within eye banks in our city. Among the weaknesses of our study, we note the lack of data systematization during data collection. Furthermore, this study represents only a fraction of the characteristics of donor tissues as it does not consider tissues from other eye banks in the city.

## Conclusions

Given the high rate of violent deaths in our region, tissue donors are predominantly young individuals, resulting in excellent endothelial cell counts and tissue viability in their microscopic studies. In the present study, there was a lower rate of tissue discard compared to similar studies, further demonstrating that in our case, most discards were due to infectious pathologies rather than tissue processing defects.

Studies of this nature provide the opportunity to identify areas for improvement in the selection and processing of tissue banks.
